# Drought Stress Priming Improved the Drought Tolerance of Soybean

**DOI:** 10.3390/plants11212954

**Published:** 2022-11-02

**Authors:** Mariz Sintaha, Chun-Kuen Man, Wai-Shing Yung, Shaowei Duan, Man-Wah Li, Hon-Ming Lam

**Affiliations:** 1 School of Life Sciences, The Chinese University of Hong Kong, Shatin, Hong Kong, China; 2 Centre for Soybean Research of the State Key Laboratory of Agrobiotechnology, The Chinese University of Hong Kong, Shatin, Hong Kong, China

**Keywords:** water content, photosynthesis, transpiration, gene expression, drought memory, physiology

## Abstract

The capability of a plant to protect itself from stress-related damages is termed “adaptability” and the phenomenon of showing better performance in subsequent stress is termed “stress memory”. While drought is one of the most serious disasters to result from climate change, the current understanding of drought stress priming in soybean is still inadequate for effective crop improvement. To fill this gap, in this study, the drought memory response was evaluated in cultivated soybean (*Glycine max*). To determine if a priming stress prior to a drought stress would be beneficial to the survival of soybean, plants were divided into three treatment groups: the unprimed group receiving one cycle of stress (1S), the primed group receiving two cycles of stress (2S), and the unstressed control group not subjected to any stress (US). When compared with the unprimed plants, priming led to a reduction of drought stress index (DSI) by 3, resulting in more than 14% increase in surviving leaves, more than 13% increase in leaf water content, slight increase in shoot water content and a slower rate of loss of water from the detached leaves. Primed plants had less than 60% the transpiration rate and stomatal conductance compared to the unprimed plants, accompanied by a slight drop in photosynthesis rate, and about a 30% increase in water usage efficiency (WUE). Priming also increased the root-to-shoot ratio, potentially improving water uptake. Selected genes encoding late embryogenesis abundant (LEA) proteins and MYB, NAC and PP2C domain-containing transcription factors were shown to be highly induced in primed plants compared to the unprimed group. In conclusion, priming significantly improved the drought stress response in soybean during recurrent drought, partially through the maintenance of water status and stronger expression of stress related genes. In sum, we have identified key physiological parameters for soybean which may be used as indicators for future genetic study to identify the genetic element controlling the drought stress priming.

## 1. Introduction

Soybean is not only an economically important oil seed crop, but it also has many other desirable attributes. As a low-cost meat alternative in a vegan diet [1] and a potential scaffolding material to produce more affordable lab-grown meat [2], it could help to alleviating the meat protein crisis in the world today. Soy proteins have anti-cholesterol and anti-carcinogenic effects as well as protective effects against diabetes, kidney disease, and menopausal hot flashes [3,4], while soy fiber has health benefits such as acting as a laxative [5]. Soybean hull can be used commercially to produce bioethanol, butanol, enzymes (cellulases, xylanases, etc.), phytohormones (gibberellin), and prebiotics, etc. [6].

However, cultivated soybean is susceptible to drought [7], a major abiotic stress. Decreased precipitation and/or increased evaporation due to climate change will increase the severity of drought stress across the world in the second half of the 21st century [8]. According to a simulation, it is predicted that the drought-driven yield loss will increase by around 16% by the end of the 21st century without any drought adaptation by the plant [9]. Fortunately, there are various mechanisms by which soybean plants adapt to drought stress [10].

Various plants are known to perform better in subsequent stress as a result of their previous exposure to the same stress. This phenomenon of achieving better performance in the subsequent stress cycle is called stress memory [11]. This phenomenon has been reported in various plants showing drought memory responses, including *Arabidopsis thaliana* [12,13,14], *Zea mays* [15], wheat, [16]*,* rice [17], *Cakile maritima* [18], *Aptenia cordifolia* [19,20], and *Silene dioica* [21]. Plants with drought memory response exhibited different physiological changes, including reduced stomatal conductance, lowered photosynthesis, as well as improved relative water content (RWC), chlorophyll content, photosystem II (PSII) efficiency, and better performance against oxidative damage, than naïve plants [15,17,18,19,20]. It was found that some “trainable memory genes” in *Arabidopsis* can be “trained” to be expressed by the plant in the first stress cycle and are then expressed at a higher efficiency when exposed to subsequent similar stresses. These trainable memory genes were found to have some “memory marks” such as higher histone 3 lysine 4 trimethylations (H3K4me3) and higher Ser5-phosphorylated RNA polymerase II accumulation in the promoter region, which are responsible for their higher expression during subsequent stresses [13,22].

Stress memory response has also been assessed in soybean. For example, salt-primed seedlings were found to have altered histone 3 lysine 4 dimethylations (H3K4me2), H3K4me3, and histone 3 lysine 9 acetylation (H3K9ac) marks throughout the genome to promote the response related to salt tolerance [23]. Soluble sugar and proline contents have been found to increase in the initial drought stress and then remain stable in the subsequent stresses to protect soybean plants from drought-induced osmotic stress [24]. The expressions of the proline synthesizing gene (*P5CS1*) and two genes encoding NAC transcription factors (related to the abscisic acid [ABA]-mediated pathway) showed changes in the subsequent drought stresses in soybean [24]. In another study using microarray analyses with a DNA chip, 392 soybean genes were found to have at least four-fold elevation in expression while 613 genes were found to have at least four-fold reduction in expression in the repeat stress treatment compared to the initial stress. The genes that showed elevated transcript levels in the subsequent stress included transcription factors, a trehalose (a disaccharide maintaining membrane fluidity) biosynthesis enzyme, Late Embryogenesis Abundant proteins, and PP2C-family proteins. The genes with reduced transcript levels during the repeated stress included those related to photosynthesis and primary metabolic pathways [25]. The drought-priming-responsive genes, including *osmotin* and the *WRKY*, *SMP*, *MYB*, *NAC*, *PP2C*, *AP2*, and *LEA* families, have been found to have very high induction during the second stress treatment [25]. In some cases, the memory obtained from the first drought stress treatment was transferable to the next generation. The transgenerational effect of drought stress was observed in soybean when the stress was experienced by the maternal line during the reproductive stage, resulting in reduced seed quality, rate of germination, and seed vigor in the F1 generation [26] and higher dehydrin protein levels in seeds [27]. From these studies, it is obvious that soybean has a drought memory response.

In soybean, most prior studies focused on the changes in metabolite concentrations and gene expressions after priming in soybean. However, whether these changes are reflected in the physiology is still unclear. Previously, we have identified a drought-sensitive soybean variety, C08, in a gradual soil-drying experiment [28]. We hypothesize that the drought stress tolerance of C08 might be enhanced through drought stress priming. The enhanced drought tolerance might be assessed through the physiological parameters. In this study, we attempted to investigate the physiological changes upon drought stress priming. To study the changes in the physiology of soybean plants involved in the drought stress memory response, C08 plants were treated once or twice with drought stress. Drought sensitivity, growth and photosynthetic performance were compared between the different treatment groups to determine the impact of drought stress memory on the plant physiology. The expressions of stress memory marker genes were also investigated. This is the first comprehensive physiological study of drought stress priming in soybean.

## 2. Results

### 2.1. Glycine Max C08 Demonstrated Drought Stress Memory

**Figure 1:**
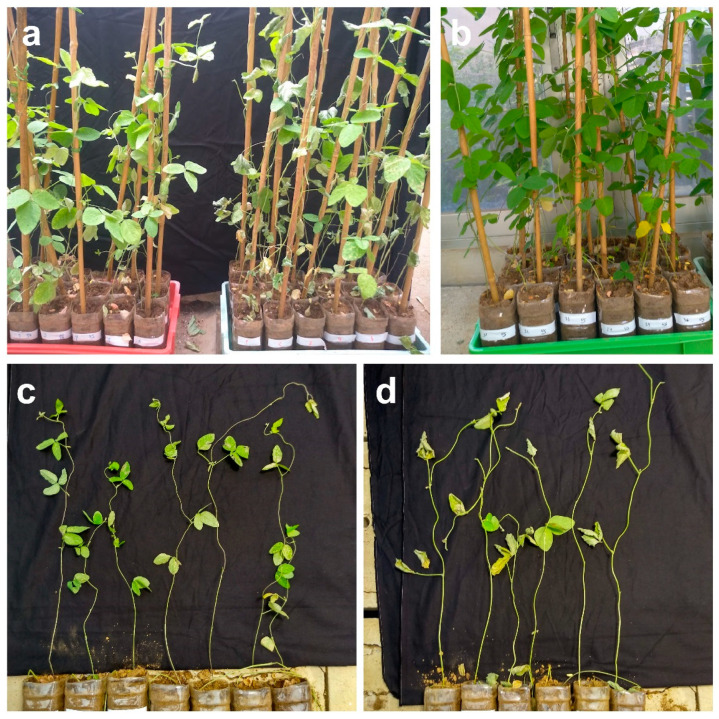
Phenotypes of the drought-sensitive soybean C08 plants after drought treatment. (**a**) Left: plants after 2 cycles of drought treatment (2S: 7 days without irrigation until the first sign of wilting, followed by 5 days of recovery and then 10 days without irrigation). Right: plants receiving only 1 cycle of drought treatment (1S: 10 days without irrigation with no priming). (**b**) Phenotype of unstressed plants (US) well irrigated throughout the experiment. (**c**) The 2S plants laid out individually on a flat surface. (**d**) The 1S plants laid out individually on a flat surface.

**Figure 2:**
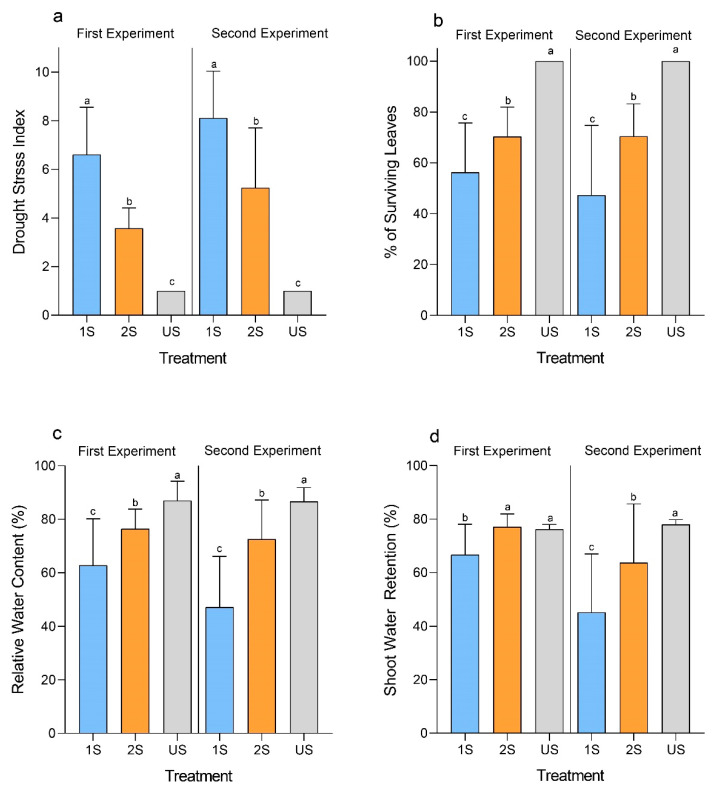
Performance of primed (2S) and unprimed (1S) soybean C08 plants under drought treatment compared to the untreated control (US). (**a**) Drought stress index, n = 28–30 plants. (**b**) Percentage of surviving leaves, n = 20–30 plants. (**c**) Relative water content, n = 19–29 plants. (**d**) Shoot water retention, n = 26–30 plants. Error bars indicate standard deviation. Wilcoxon rank-sum test was used to compare between the mean values of each treatment following one-way ANOVA. Different letters above the bars indicate significant differences between groups at *p* < 0.05. Each experiment was performed twice (First and Second Experiment), with similar results.

The soybean cultivar C08 has previously been demonstrated to be a drought-sensitive variety [28]. To determine the effect of drought stress memory on these plants, the seedlings of soybean C08 were treated with drought stress once (unprimed, 1S) or twice (primed, 2S). For the 2S treatment group, a recovery period was given to the plants in between the two drought treatments. Well-watered plants were used as control (unstressed, US). The detailed treatment design can be found in Figure S1. In two independent experiments, the drought-treated plants were suffering from different degrees of drought damage (Figure 1). We quantitatively assessed the overall performance of the plants using the drought stress index (DSI), and percentage of surviving leaves. The US plants had a DSI = 1, meaning that all the plants were healthy at the end of the experiments (Figure 2a). On the contrary, the soybean plants under 1S treatment had a significantly higher DSI compared to the 2S treatment group (Figure 2a). Similarly, the soybean plants under 1S treatment had a lower percentage of surviving leaves than those under 2S treatment (Figure 2b). Both the DSI and percentage of surviving leaves indicated that the soybean plants receiving repeated drought treatments performed better than those receiving only a single treatment. The first drought treatment may have primed the plants for responding to the subsequent stress.

### 2.2. Priming for Drought Stress Resulted in Better Water Retention Abilities than in Unprimed Plants

A better water retention ability could improve the survivorship of a drought-stressed plant. To determine whether the plants receiving repeated drought treatments gained a better water retention ability, the relative water content (RWC) and the shoot water content were assessed.

Relative water content (RWC) is a commonly used parameter for measuring the amount of water retained by a leaf. We measured the RWC of the middle leaflet of the top trifoliate leaves. Both the 1S and 2S treatments led to a significant reduction in RWC in these plants compared to the US control (Figure 2c). However, consistent with the results on DSI and percentage of surviving leaves, the RWC of the 1S plants (62.77% ± 17.42) was significantly lower than that of the 2S plants (76.38% ± 7.36). A similar trend was also observed in another independent experiment, with a significant difference between the two treatment groups (Figure 2c).

Furthermore, we assessed the shoot water content under the three treatment conditions. The single drought (1S) treatment led to a significant drop in the shoot water content (Figure 2d). In contrast, the plants receiving two drought treatments (2S) were able to maintain a significantly higher shoot water content than those in the 1S group. That means the primed plants were able to maintain a better water status in the subsequent stress.

**Figure 3:**
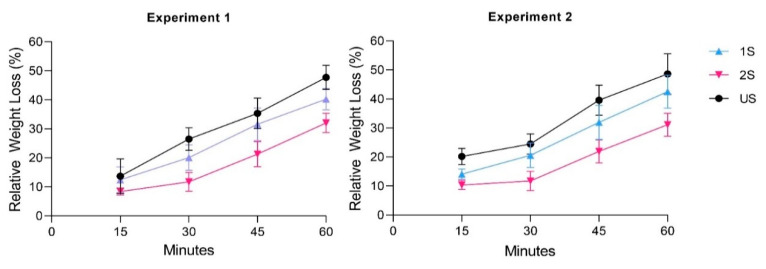
Rates of fresh weight loss over an hour from a detached leaflet of the top trifoliate leaves of primed (2S), unprimed (1S) and unstressed control (US) soybean C08 plants relative to the initial fresh weight immediately after detachment. n = 12 plants. Error bars indicate standard deviation. The experiment was performed twice (Experiments 1 and 2), with similar results.

We speculated that a lower rate of water loss from the leaves was the cause of a better RWC and shoot water content. Hence, the rate of water loss from a detached leaflet of the top trifoliate leaf was examined. Although the rate of water loss from the leaves of the 2S and 1S plants was similar at 15 min after detachment (Figure 3), the differences became larger as time went on. The leaves from the 1S plants lost significantly more water than those from the 2S plants. In both independent experiments, the 1S plants had around 40% water loss in terms of relative weight loss within 60 min of leaf detachment while the 2S plants lost only around 30%. This suggested that the 2S plants were better at reducing water loss than the 1S plants.

### 2.3. Drought Stress Memory Reduced the Rates of Photosynthesis, Transpiration and Stomatal Conductance and Increased Water Usage Efficiency

**Figure 4:**
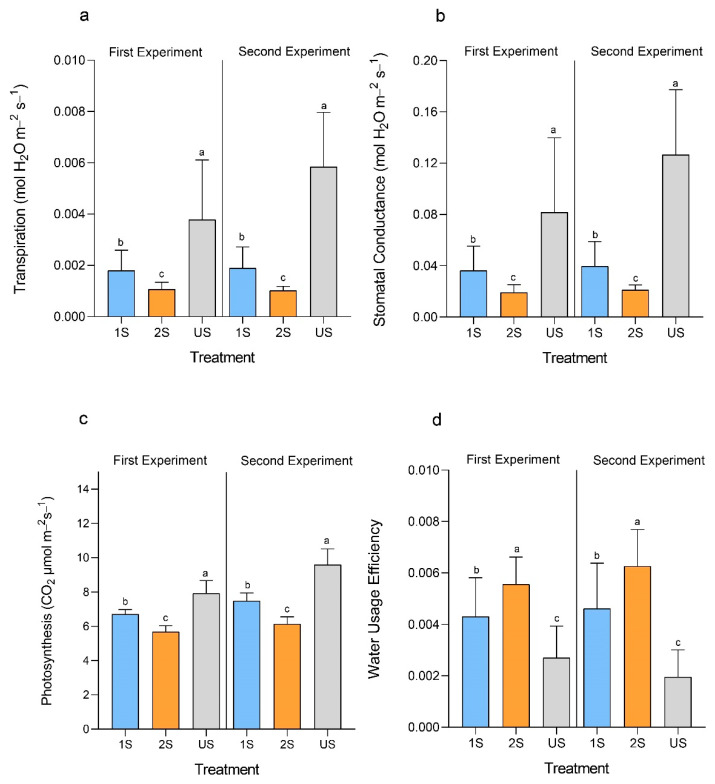
Changes in the photosynthesis-related parameters of primed (2S) and unprimed (1S) soybean C08 plants under drought treatment compared to the unstressed control (US). (**a**) Rate of transpiration, n = 12 plants. (**b**) Stomatal conductance, n = 12 plants. (**c**) Rate of photosynthesis, n = 12 plants. (**d**) Water usage efficiency, n = 12 plants. Error bars indicate standard deviation. Wilcoxon rank-sum test was used to compare between the mean values of each treatment following one-way ANOVA. Different letters above the bars indicate significant differences between groups at *p* < 0.05.

Under drought conditions, the photosynthetic functions of plants are largely hampered due to the closure of stomata for water conservation. Thus, we investigated the effect of drought stress priming on photosynthesis. As expected, the drought treatments have reduced the rates of photosynthesis, transpiration rate and stomatal conductance of plants in both treatment groups (1S and 2S) when compared to the well-watered controls (US) (Figure 4a–c). Furthermore, plants in the 2S group had a significantly lower transpiration rate and stomatal conductance accompanied by a slightly lower photosynthetic rate than those in the 1S group.

By lowering the transpiration rate to conserve water, photosynthesis is slowed down, which in turn hampers plant growth. A high instantaneous water usage efficiency (WUEi), expressed as the ratio of the rate of photosynthesis to the rate of transpiration, would infer that the plant has maintained a high photosynthetic rate and/or a low rate of water loss through transpiration. Drought priming reduced both photosynthesis and transpiration in soybean plants but increased the WUEi. The drought treatments improved the WUEi of the plants from both 1S and 2S groups compared to those from the US group (Figure 4d), as the drop in the transpiration rate was more prominent than the reduction in photosynthesis. Moreover, the primed 2S soybean plants demonstrated an even better WUEi than the 1S soybean plants, suggesting the plants in the 2S treatment group were more adapted to the drought stress.

**Figure 5:**
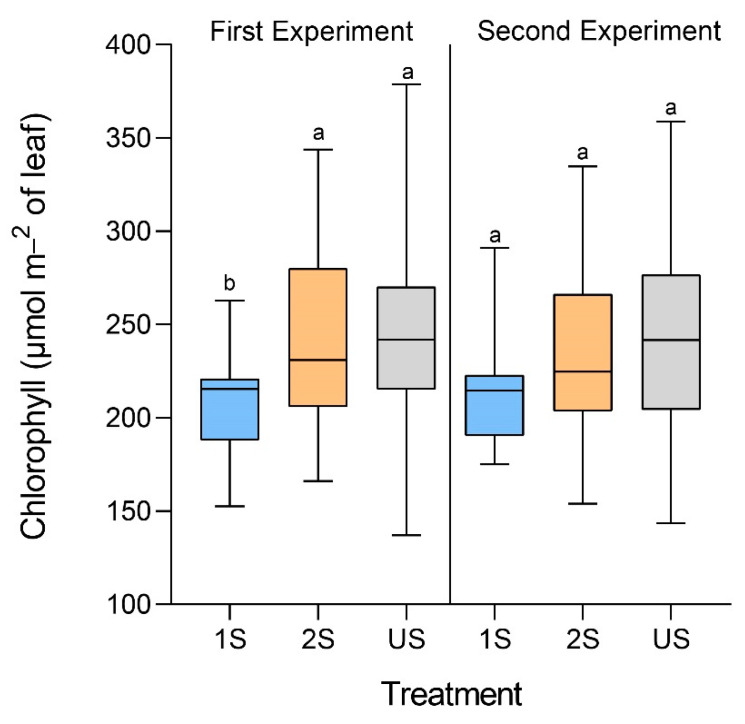
Box-and-whisker plots of the chlorophyll contents of primed (2S) and unprimed (1S) soybean C08 plants under drought treatment compared to the unstressed control (US). The whiskers represent the maximum and minimum values in the sample. Wilcoxon rank-sum test following one-way ANOVA was used to compare between the mean values of each treatment. Different letters indicate significant differences between groups at *p* < 0.05. n = 19–30.

Next, we investigated whether the drop in photosynthesis was due to the disintegration of chlorophyll under the stress conditions. Although there was a slight drop in the chlorophyll content in the plants from the 1S treatment group compared to those from the US control group, the difference was only statistically significant in one of the two independent experiments (Figure 5). In contrast, there was no significant difference in chlorophyll contents between the plants from the 2S treatment group and the US control group in either experiment.

### 2.4. Drought Stress Memory Did Not Affect Growth Performance beyond the Effects of Drought Stress Itself

Water deficiency can lead to the diminished growth of plants. As expected, the plants from the 1S and 2S treatment groups had significantly lower shoot and root weights than those from the US group (Table 1). However, interestingly, although the 2S plants received drought treatment twice and the 1S plants were treated only once, there was no significant difference in shoot and root lengths or shoot and root weights between the 1S plants and the 2S plants. Furthermore, the maximum root length of the 2S plants was the longest among the three treatment groups (Table 1), which also led to a significant increase in the root-length-to-shoot-length ratio (Table 1).

### 2.5. Drought Stress Memory Induced the Expressions of Selected Drought Priming-Responsive Genes

**Figure 6:**
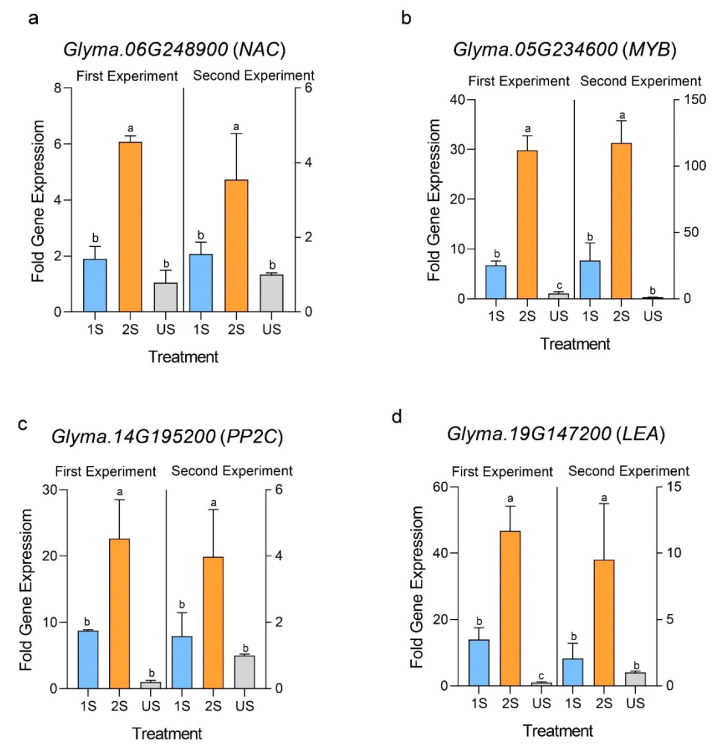
The relative expression levels of selected genes in primed (2S) and unprimed (1S) soybean C08 plants under drought treatment and the unstressed control (US) were analyzed by RT-qPCR. (**a**) Expression of Glyma.06G248900, (**b**) Glyma.05G234600, (**c**) Glyma.14G195200, and (**d**) Glyma.19G147200 was calculated by 2^−ΔΔCT^ method. The data are presented as the mean of three technical replicates ± SD. Tukey’s honest significance test was used to compare between the mean values of each treatment following one-way ANOVA. Different letters above the bars indicate significant differences between groups at *p* < 0.05. *act11* and *elf1b* were used as the reference genes. The scale on the left *y*-axis refers to the data from the first experiments and that on the right *y*-axis refers to those from the second experiment.

The expressions of selected transcription factor genes related to drought stress responses were analyzed using RT-qPCR. Four putative candidate genes were selected from among the drought priming-responsive genes from previous drought stress studies on soybean [25]. These genes all showed significantly higher induction in the group with priming (2S) compared to the unprimed group (1S) in two independent experiments (Figure 6).

## 3. Materials and Methods

### 3.1. Plant Materials, Growth and Treatment Conditions

Seeds of *Glycine max* C08 (cultivar name: Union) [29] were germinated in vermiculite in a greenhouse at the Chinese University of Hong Kong (22°25′7″ N 114°12′26″ E), Shatin, Hong Kong, China. Five-day-old healthy seedlings were transplanted in modified plastic soft drink bottles filled with sandy loam soil and peat moss in a 1:1 ratio [28]. The tops of the plastic bottles were removed and flapping windows were made for inserting a soil moisture probe for monitoring the soil moisture over time. Five holes were drilled at the bottom of each bottle. The window flaps were sealed with water-resistant tape to prevent water loss through evaporation when the probe was removed. The plants were allowed to grow until the first trifoliate leaf was fully open at an average monthly temperature of 25 °C and average air humidity of 69%. During this period, the plants were sub-irrigated with the same amount of water (30 mL/plants/day) [28], and were divided into three groups: unstressed (US) control, once-stressed (1S), and twice-stressed (2S). Each group began with 30 seedlings to maximize the statistical power [30]. After the first trifoliate leaf was fully open, the first drought cycle was applied to the 2S group by withdrawing the irrigation [28]. The soil moisture content of the 2S group was measured with a soil moisture meter (TZS-W, China) every other day through the window on the bottle until the reading dropped to 8 (after 7 days). The probe reading of 8 corresponds to 35% of field capacity and the plants showed the sign of wilting at this point in a preliminary study. Then irrigation was resumed on the next day to allow the plants to recover for 5 days. After the recovery period, irrigation water was withdrawn from both 1S and 2S treatment groups, while the US treatment group was kept well-watered for a 10-day period. The experiment was repeated twice. The treatment scheme is depicted graphically in Figure S1.

### 3.2. Determination of Drought Stress Index (DSI)

The DSI was determined according to the visual assessment scheme [31] with some modifications (Figure S2). As the plants used in this experiment have around five nodes, a 10-point scale was used to properly differentiate the phenotypes between the plants (Figure S2). The plants showing no symptoms of stress were scored 0 whereas the dead plants scored 10. Scoring was done to all plants at the end of the second drought treatment of the 2S plants.

### 3.3. Determination of the Growth-Related Parameters

At the end of the second stress cycle, the number of nodes, total number of leaves, and number of surviving leaves of all plants were counted. Maximum root length and shoot lengths were measured using a ruler. The fresh weight of the shoot was measured using an analytical balance. The roots were gently washed with tap water and both shoots and root were dried in an oven at 65 °C for 72 h before measuring the biomass. The percentage of surviving leaf was calculated using this formula:

$$\mathrm{Percentage}\;\mathrm{of}\;\mathrm{surviving}\;\mathrm{leaf}\;=\frac{\mathrm{No}.\;\mathrm{of}\;\mathrm{surviving}\;\mathrm{leaves}}{\mathrm{Total}\;\mathrm{number}\;\mathrm{of}\;\mathrm{leaves}}\times 100\%$$

### 3.4. Determination of the Relative Water Content (RWC)

The middle leaflet of the topmost trifoliate leaves of the treated plants were harvested for the determination of RWC. Upon collection, the leaves were immediately sealed in plastic zip bags and placed on ice to prevent water loss. After measuring the fresh weight, the leaves were soaked in tap water in the zip bags. The turgid weights of the fully saturated leaves were measured after soaking for 24 h. The leaves were then dried in an oven at 65 °C for 48 h for the measuring the leaf dry weight. RWC was calculated using this formula:

$$\mathrm{RWC}=\frac{\mathrm{FW}-\mathrm{DW}}{\mathrm{TW}-\mathrm{DW}}\times 100\%$$

where FW, DW, and TW were the fresh weight, dry weight, and turgid weight of the leaflet, respectively.

### 3.5. Calculation of the Shoot Water Content

After treatment, the entire above-ground parts of the treated plants were collected. After measuring the fresh weight, the plant materials were dried in an oven at 65 °C for 72 h to determine the dry weight. The shoot water content was calculated in relation to the fresh weight with this formula [32]:

$$\mathrm{Shoot}\;\mathrm{water}\;\mathrm{content}=\frac{\mathrm{SFW}-\mathrm{SDW}}{\mathrm{SFW}}\times 100\%$$

where SFW was the shoot fresh weight and SDW was the shoot dry weight. Shoot referred to all the above-ground tissues including stems and leaves.

### 3.6. Measurement of Photosynthesis-Related Parameters

The topmost trifoliate leaves from each treatment group were selected for the measurement. Within each treatment group, plants with similar DSI were selected for the measurement. The rates of photosynthesis, transpiration and stomatal conductance were measured using the LI-COR 6800 portable photosynthesis system between 9:00 a.m. to 12:30 p.m. local time with a photosynthetic photon flux density of 800 μmol m^−2^ s^−1^, CO_2_ concentration at 395–400 μmol mol^−1^, and 40% leaf chamber air humidity at the ambient air temperature of 25 °C. The average of two measurements taken from the same position on the top trifoliate leaves of each selected plant was used. Instantaneous Water Usage Efficiency (WUEi) was calculated by dividing the ratio of photosynthesis (after converting the µmol CO_2_ to mol CO_2_) by the rate of transpiration. Measurements were taken at the end of the second drought stress (10th day).

Chlorophyll contents of the middle leaflet of the trifoliate leaves were measured using a chlorophyll concentration meter (MC 100, Apogee, Logan, UT, USA). The measurement was taken between 9:00 a.m. to 12:30 p.m. local time from all surviving plants at the end of the second drought stress.

### 3.7. Measurement of Rate of Water Loss (RWL) from Leaves

The plants used for measuring gaseous exchange were also used for measuring the rate of water loss. One leaflet was detached from the top trifoliate leaf and the fresh weight was measured immediately. The leaflets were then left uncovered at room temperature and humidity and their weight was taken every 15 min using an analytical balance. The percentage weight loss of each detached leaflet from its initial fresh weight was calculated for each time point.

### 3.8. RT-qPCR Analyses

Total RNA was extracted from the top two trifoliate leaves using TRIzol reagent. Samples were collected from 3 plants from each treatment group. After that, DNase I treatment was performed to eliminate DNA contamination prior to converting the RNA to cDNA using iScript™ cDNA Synthesis Kit (Bio-Rad, Hercules, CA, USA, cat# 1708890) according to the manufacturer’s protocol. Gene expressions were then analyzed by quantitative PCR using SsoAdvanced Universal SYBR Green Supermix (Bio-Rad, Hercules, CA, USA, cat# 172-5271). The polymerase chain reaction was carried out using a CFX384 Touch Real-Time PCR Detection System (Bio-Rad, Hercules, CA, USA). Fold changes in expression were calculated by the 2^−ΔΔCt^ method using *act11* and *elf1b* as reference genes [33,34]. For the target genes, the specific primers were designed spanning the exon-intron junction using the primer-BLAST tool (NCBI) to prevent the amplification of genomic DNA. These primer sequences can be found in Table S1.

### 3.9. Statistical Analysis

Data from the 1S, 2S, and US groups were compared by one-way ANOVA by rank (Kruskal-Wallis test). Then post hoc pairwise comparisons were done by Wilcoxon rank-sum test (non-parametric) using the R programming and Graphpad Prism software version 8.0.0 (San Diego, California, USA, www.graphpad.com). Fold changes in gene expression were compared by Tukey’s honest significance test following one-way ANOVA. The differences were considered to be significant at *p* < 0.05.

## 4. Discussion

Stress memory refers to the ability of pre-stressed plants to gain higher tolerance towards the subsequent stress, compared to naïve plants experiencing stress for the first time. Several studies have investigated drought stress memory response in soybean. For example, the changes in soluble sugar and protein concentrations [24], and gene expressions [25] in drought stress-primed soybean have been evaluated. Furthermore, potential transgenerational drought stress memory has also been delineated [26,27]. Here we have added to the understanding of drought stress memory by evaluating the physiological responses of soybean to drought stress priming.

In our study, primed (2S) plants showed physiological adaptation and less severe symptoms of drought than unprimed plants (1S) due to the presence of stress memory.

Roots can sense the dryness of the soil and send a signal, e.g., ABA, to the shoot as the earliest warning message to close the stomata [35]. Faster declination of stomatal conductance, rate of transpiration, and a lower rate of water loss from the detached leaf under drought stress are the traits associated with a more drought-tolerant soybean genotype [28,36]. In our study, the primed plants had much lower stomatal conductance and rate of transpiration, indicating more tolerance of the drought. This might have been achieved by better root-to-shoot communication, or a higher sensitivity towards ABA signal. In potato plants, drought priming induced a thicker cuticular layer [37] which is inversely proportional to water loss from the detached leaf [38]. In our study, a lower rate of water loss from the detached leaf in the primed plants might have been achieved by rapid stomatal closure or thicker cuticle.

Root-to-shoot ratio increases during drought stress in various plants [39,40] and it is associated with a more drought-tolerant genotype in soybean [41]. As the root-to-shoot ratio increases during each drought stress cycle, primed plants receiving two cycles of stress had a higher root-to-shoot ratio. This helped the primed group to exploit the water resources deeper in the soil. Similar to other studies on various plants [13,15,42], primed plants also showed higher water content in our study. It was achieved by the tighter control of stomata as well as a higher root-to-shoot ratio as discussed above. Higher water content reduced wilting and leaf death resulting in a lower drought stress index and a higher percentage of alive leaves in the primed group.

Lower total chlorophyll content was expected in the primed group due to exposure to drought stress twice. However, soybean plants underwent a rapid increase in plant height and overcompensated the chlorophyll content during the recovery phase between two stress cycles [43]. Thus, in the two repeats of our study, primed plants had similar/higher chlorophyll content compared to unprimed plants. Similarly, no significant difference between total chlorophyll content in the primed and unprimed group is also observed in other plants [20,21]. This was achieved by either overcompensation of chlorophyll content during the recovery phase or the less damaging effect of drought on photosynthetic machinery in the primed plants. The mechanism for chlorophyll overcompensation is not well understood. We speculate that the priming stress enhanced the production of chlorophyll while preventing it from disintegration. For example, we observed a strong induction of the LEA protein-encoding gene in the second stress cycle. LEA protein overexpressing transgenic lines had lower ROS, higher total chlorophyll content, and higher drought tolerance in other plants [44,45,46]. In soybean, lower ROS content and higher chlorophyll content are associated with a more tolerant genotype [47]. LEA proteins are known to scavenge ROS [46]. Thus, higher induction of LEA protein might have contributed to reducing ROS’s damaging effect on chlorophyll in the primed group.

In plants, drought-induced stomatal closure to retain water leads to a lower rate of photosynthesis. It is therefore important to have a good balance between water retention and plant growth. Soybean plants achieve high WUE during moderate drought, then WUE declines when soil is further dried [48]. In our study, priming caused the plants to retain higher WUE in drier soil, while unprimed plants failed. This resulted in lower water usage per photosynthetic carbon assimilation. Consequently, despite receiving stress twice, the primed group did not have significantly lower root and shoot weight than the unprimed group. The compensation by rapid growth during the recovery phase between two stress cycles might have also contributed to this [43].

Drought stress priming also led to strong responses at the transcriptional level. The RT-qPCR analyses showed that the selected genes, *PP2C* (Glyma.14G195200), *MYB* (Glyma.05G234600), *NAC* (Glyma.06G248900), and *LEA* (Glyma.19G147200) had higher expressions in the primed plants compared to the unprimed plants. In a previous study, microarray analyses revealed higher transcript levels of all these genes during the second drought stress compared to the first stress in soybean [25], which is consistent with our qPCR analyses. The stronger response of the gene expression could potentially be explained by the chromatin remodeling and the global change in histone modification during the priming stress [23]. ABA is known to induce pathways that mediate the conversion of *PP2C* chromatins from a repressive to an active state that can create epigenetic memory [49]. ABA-PP2C pathway mediates stomatal closure during drought stress [50]. *PP2C* (Glyma.14G195200) has been found to have a higher expression in drought-tolerant transgenic lines overexpressing the ABA biosynthesizing gene [51]. Thus, higher expression of this *PP2C* gene in primed plants is an indication that primed plants had higher sensitivity toward the drought stress-induced ABA and expected to have a rapid stomatal closure. A previous study demonstrated that transgenic soybean overexpressing *MYB* (Glyma.05G234600) showed better tolerance to drought, higher proline (osmoprotectant) contents, longer primary root, and higher induction of other genes related to drought (such as RD22B) [52]. Higher induction of this *MYB* gene in primed plants might have resulted in a higher primary root length. *LEA* genes including Glyma.19G147200 are already reported to be linked with drought memory response in soybean [25] and other plants [42]. In our study, higher *LEA* gene expression might have helped primed plants stabilize macromolecules during dehydration stress [53]. *NAC* (Glyma.06G248900) is an ortholog of A. thaliana *AtRD26* that contains a cis-regulatory element for *MYB*, *ABRE*, and *DREB*, etc. [54]. It is highly expressed in response to polyethylene glycol (PEG), abscisic acid (ABA), and drought [55], and is one of the most highly expressed *NAC*s in soybean during drought stress (known as NAC073) [56]. Higher expression of this *NAC* gene indicates better induction of interconnected drought-responsive pathways in the primed group.

Our observation suggested that drought stress priming mainly enhanced the drought avoidance of the soybean plant, through reducing water loss by being either a water-acquirer/seeker or a water-saver without much compromise in growth.

## 5. Conclusions

Drought priming significantly improved the drought stress response in the drought-sensitive soybean genotype C08 through enhancing its drought avoidance mechanisms. Furthermore, drought priming also induced the expressions of drought tolerance-related genes, leading to a more effective drought stress response. The percentage of live leaves was significantly increased while the Drought Stress Index was decreased, resulting in stronger and healthier plants.

## Figures and Tables

**Table 1:** Growth parameters of the drought-treated soybean plants.

Exp.	Group	Shoot Weight	Root Weight	Shoot Length (SL)	Root Length (RL)	Node Number	RL to SL Ratio ^#^
	1S	0.246 ± 0.12 ^b^	0.074 ± 0.06 ^b^	47.5 ± 18.8 ^b^	13.1 ± 2.9 ^ab^	5.3 ± 0.9 ^b^	0.3 ± 0.2 ^b^
1	2S	0.236 ± 0.11 ^b^	0.094 ± 0.06 ^ab^	41.5 ± 18.7 ^b^	15.3 ± 4.9 ^a^	5.6 ± 0.8 ^b^	0.4 ± 0.2 ^a^
	US	0.554 ± 0.29 ^a^	0.170 ± 0.14 ^a^	68.5 ± 27.9 ^a^	11.8 ± 2.1 ^b^	6.4 ± 1.5 ^a^	0.2 ± 0.1 ^c^
	1S	0.310 ± 0.09 ^b^	0.090 ± 0.06 ^b^	53.7 ± 16.4 ^b^	14.2 ± 3.3 ^b^	5.9 ± 0.7 ^b^	0.3 ± 0.1 ^b^
2	2S	0.272 ± 0.12 ^b^	0.108 ± 0.06 ^b^	48.8 ± 15.7 ^b^	16.9 ± 3.2 ^a^	6.0 ± 0.9 ^b^	0.4 ± 0.2 ^a^
	US	0.580 ± 0.13 ^a^	0.169 ± 0.08 ^a^	76.3 ± 14.3 ^a^	12.6 ± 2.0 ^c^	6.9 ± 0.5 ^a^	0.2 ± 0.03 ^c^

Note: 1S, unprimed; 2S, primed; and US, unstressed soybean plants. Values are expressed as mean ± standard deviation. Letters beside the values indicate significant differences between groups (*p* < 0.05). Pairwise comparisons between groups were done by Wilcoxon rank-sum test following one-way ANOVA. The weights are expressed in grams and length in centimeters. ^#^ root-length-to-shoot-length ratio. The experiment (Exp.) was performed twice.

## Data Availability

All data generated in this study are available within this manuscript and the companion Supplementary Materials.

## Supplementary Materials

The following supporting information can be downloaded at: https://www.mdpi.com/article/10.3390/plants11212954/s1, Figure S1: Experimental design, Figure S2: Drought stress index, Figure S3: Summery discussion graph, Table S1: Primer sequences.
