# Supplementary material for: Drought Stress Priming Improved the Drought Tolerance of Soybean
Source: Plants (Basel). 2022 Nov 2;11(21):2954. doi: 10.3390/plants11212954 (PMC9653977; doi:10.3390/plants11212954)
Supplement: Supplementary file 1 [file plants-11-02954-s001.zip › Figure S1.pdf]

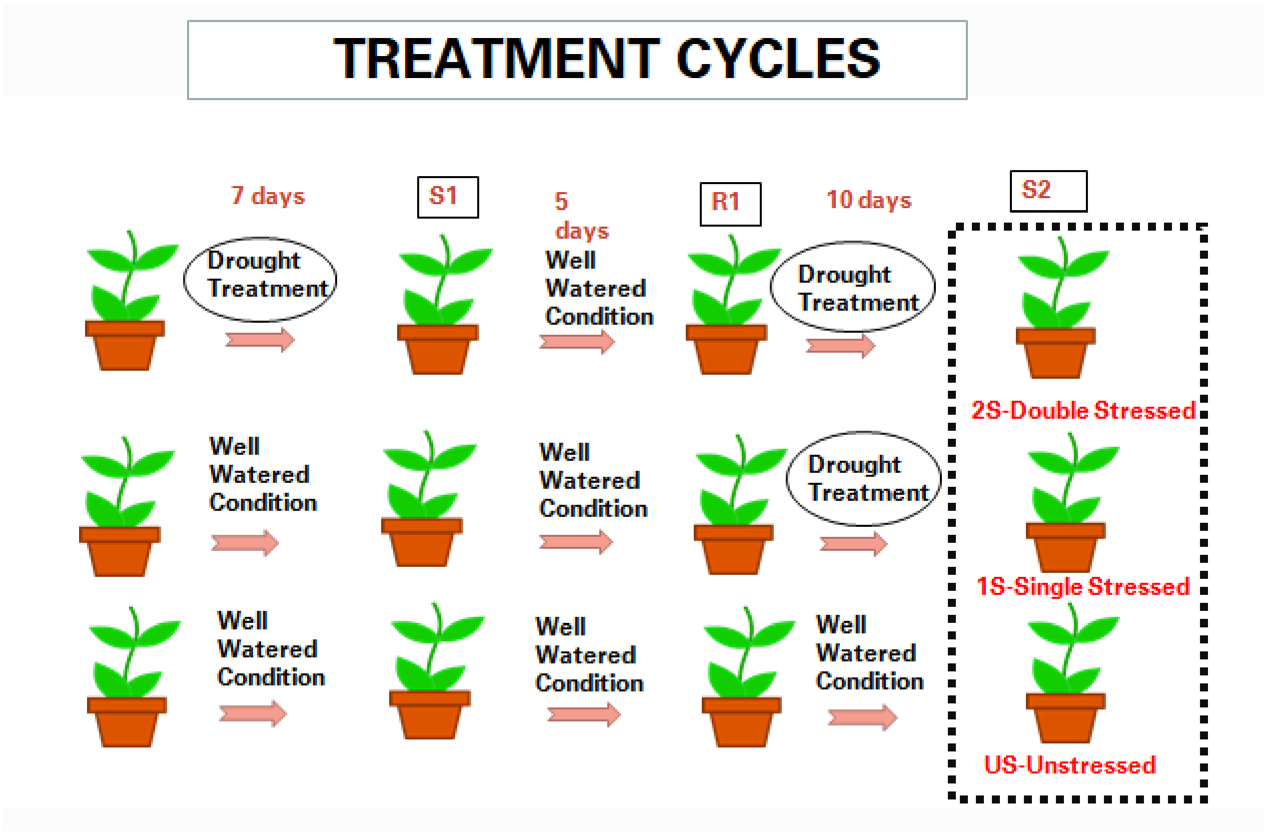

Figure S1. The plants were grown inside a greenhouse in plastic bottles. On day 15, the first stress cycle was applied to the 2S group by withdrawing water. After 7 days of stress, the water supply was resumed for 5 days to allow the plants to recover. After recovery, a 10-day-long stress cycle was applied to both the primed 2S group and the unprimed 1S group. All data were collected on the 10th day. A well-watered condition was maintained for the unstressed (US) control group throughout the experiment.
