# Supplementary material for: Drought Stress Priming Improved the Drought Tolerance of Soybean
Source: Plants (Basel). 2022 Nov 2;11(21):2954. doi: 10.3390/plants11212954 (PMC9653977; doi:10.3390/plants11212954)
Supplement: Supplementary file 1 [file plants-11-02954-s001.zip › Figure S2.pdf]

DSI	Description
1	Full fresh
2	Very slight loss of vigor
3	Slight loss of vigor (softness felt), no leaf rolling
4	1 trifoliate leaf is rolled
5	>1 trifoliate leaves are rolled
6	All leaves are rolled, but no dead leaf
7	1 trifoliate leaf is dead/crispy
8	>1 trifoliate leaves are dead/crispy
9	Full plant dead except 1 trifoliate leaf (all leaves crispy except 1TF)
10	Full dead (all leaves are crispy)

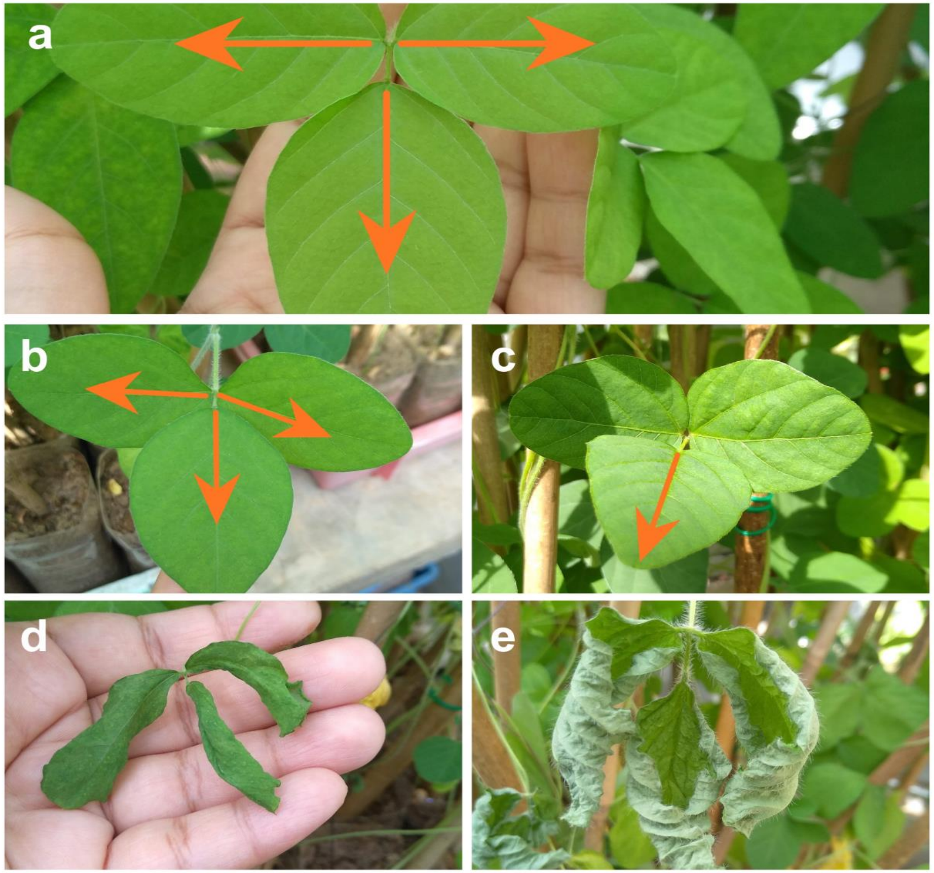

Figure S2. Left panel: detailed description of the drought stress index scoring scheme. Right panel: Picture showing the different status of leaves that correspond to the scoring. a. turgid leaf (score 1), b. leaf with very slight loss of vigour (score 2), c. leaf with slight loss of vigour (score 3), d. leaf rolling (score 4) and e. crispy leaf (score 7).
