# Supplementary material for: Drought Stress Priming Improved the Drought Tolerance of Soybean
Source: Plants (Basel). 2022 Nov 2;11(21):2954. doi: 10.3390/plants11212954 (PMC9653977; doi:10.3390/plants11212954)
Supplement: Supplementary file 1 [file plants-11-02954-s001.zip › Figure S3.pdf]

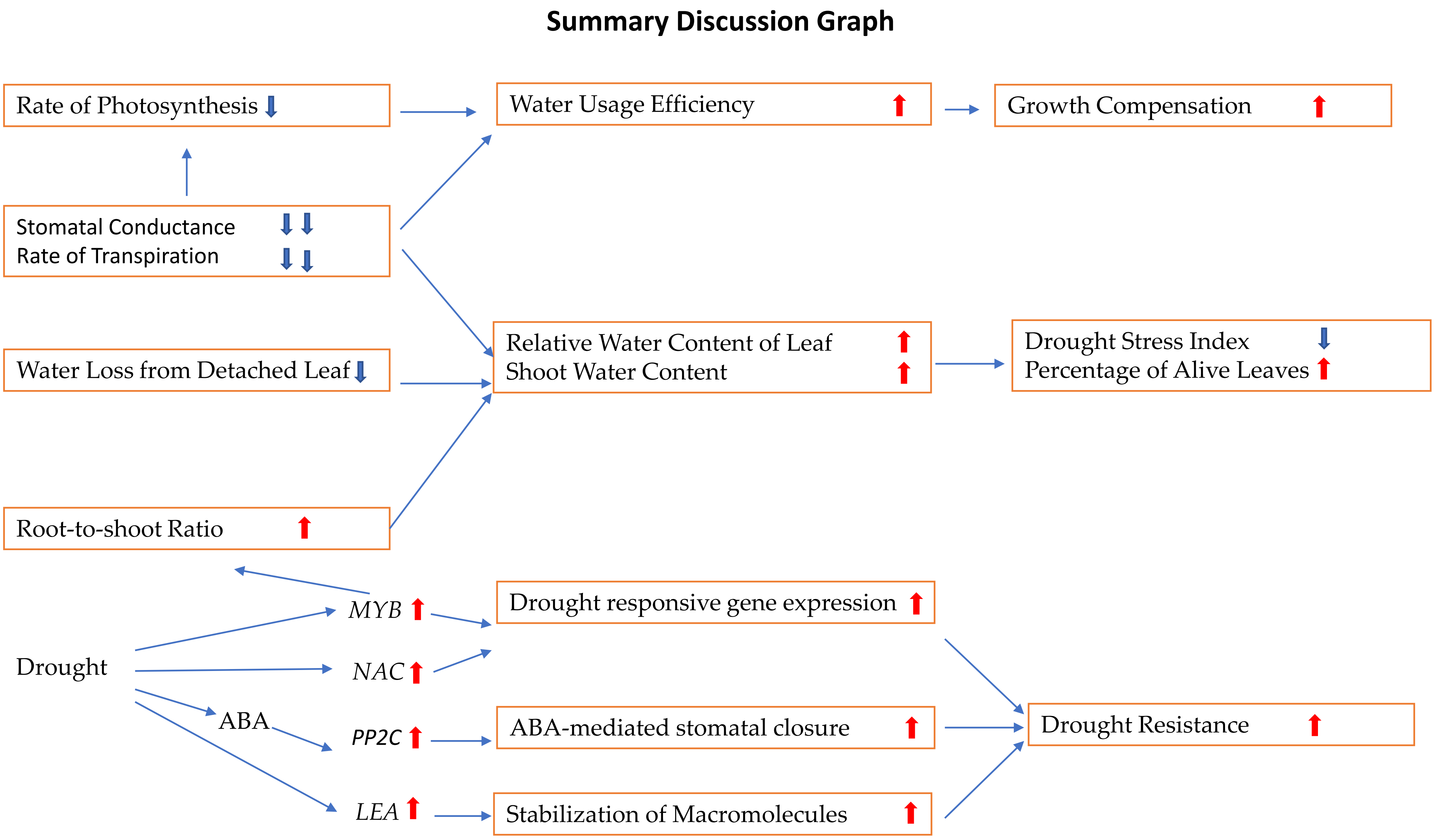

Figure S3. Priming elicits a more effective drought stress response by drought avoidance and expressions of drought tolerance-related genes.
