# Supplementary material for: Drought Stress Priming Improved the Drought Tolerance of Soybean
Source: Plants (Basel). 2022 Nov 2;11(21):2954. doi: 10.3390/plants11212954 (PMC9653977; doi:10.3390/plants11212954)
Supplement: Supplementary file 1 [file plants-11-02954-s001.zip › Table S1.pdf]

Table S1. Primer sequences used in this study

Gene		Primer Sequence (5′-> 3′)
Glyma.06G248900-1 (*NAC*)	Forward primer	GGAACCGCAAAGCTTGATGA
	Reverse primer	TGCTGCATTCCATAGCCAAA
Glyma.05G234600-1 (*MYB*)	Forward primer	ATATTTCTAGTCATGGAGAAGGGC
	Reverse primer	TTCCCAGTTCGCTTTAATCCTGA
Glyma.14G195200-1 (*PP2C*)	Forward primer	GATGGATTATGGGATAAGGTTAGT
	Reverse primer	ACTTGTGTCATCCAAAGAGC
Glyma.19G147200-1 (*LEA*)	Forward primer	ACTGTCCAAGAGAAGGCAGAA
	Reverse primer	CTCCGCCTGGTTCATCTTTG
elf1b	Forward primer	CCACTGCTGAAGAAGATGATGATG
	Reverse primer	AAGGACAGAAGACTTGCCACTC
act11	Forward primer	CGGTGGTTCTATCTTGGCATC
	Reverse primer	GTCTTTCGCTTCAATAACCCTA
